# Anstey A. Under the Skin – A Dermatologist's Fight to Save the NHS London: Whitefox Publishing Ltd; 2022

**DOI:** 10.1111/ajd.13914

**Published:** 2022-09-09

**Authors:** Alex J Chamberlain

**Affiliations:** ^1^ Central Clinical School Monash University, Alfred Health Prahran Victoria Australia; ^2^ Victorian Melanoma Service Alfred Health Melbourne Victoria Australia

*Under the skin* is an intricately woven tale of Professor Alex Anstey's career as a junior doctor then general practitioner and ultimately academic dermatologist working in the NHS. Personal memoirs are punctuated by amusing clinical vignettes, key moments in recent British political history and chapters devoted to heroes in health care. These influential and inspirational men and women range from military nurses to the founding father of evidence‐based medicine to pioneering Oxford Regius Professors and giants of medical education. These heroes have been well researched, and the quirky details the author has uncovered will delight readers.

The book provides valuable insights into the history, the machinations and struggles of the NHS both in the early days and during the pandemic. Anstey details his inspiration and journey to improve clinical efficiency in his corner of Wales. In 2000, his efforts ultimately led the Gwent Healthcare NHS trust in winning the UK Dermatology Team of the Year. The foundation of repairing a strained hospital system he details is based on consultation with all team members and connecting with the local GPs through personal interactions, education, triage and telehealth. This is a thoughtful book on how health services run and how clinicians must be in tune with both their support team and their managers, with lessons applicable beyond dermatology.

Other chapters are devoted to academia, research, scientific journal stewardship and politics. Contemporary themes of teledermatology (even more relevant post‐COVID‐19) and patient partnership or empowerment are discussed. The charming Welsh personalities and stories only augment the tale. I enjoyed this beautifully written book very much and would recommend it to not only dermatologists but all healthcare workers with an interest in hospitals and health management. If you have worked in the NHS or aspire to building a better team, equally this is essential reading.

## CONFLICT OF INTEREST

No conflict of interest to declare.

